# Simultaneous Quantification of Chloramphenicol, Thiamphenicol, Florfenicol, and Florfenicol Amine in Animal and Aquaculture Products Using Liquid Chromatography-Tandem Mass Spectrometry

**DOI:** 10.3389/fnut.2021.812803

**Published:** 2022-01-13

**Authors:** Hae-Ni Jung, Da-Hee Park, Yeon-Jae Choi, Se-Hyeong Kang, Hee-Jung Cho, Jeong-Min Choi, Jae-Han Shim, Ahmed A. Zaky, A. M. Abd El-Aty, Ho-Chul Shin

**Affiliations:** ^1^Department of Veterinary Pharmacology and Toxicology, College of Veterinary Medicine, Konkuk University, Seoul, South Korea; ^2^Natural Products Chemistry Laboratory, College of Agriculture and Life Sciences, Chonnam National University, Gwangju, South Korea; ^3^Department of Food Technology, National Research Centre, Cairo, Egypt; ^4^Department of Pharmacology, Faculty of Veterinary Medicine, Cairo University, Giza, Egypt; ^5^Department of Medical Pharmacology, Medical Faculty, Ataturk University, Erzurum, Turkey

**Keywords:** chloramphenicol, thiamphenicol, florfenicol, florfenicol amine, residue analysis, LC-MS/MS, method development

## Abstract

The accumulation of antimicrobial residues in edible animal products and aquaculture products could pose health concerns to unsuspecting consumers. Hence, this study aimed to develop a validated method for simultaneous quantification of chloramphenicol (CAP), thiamphenicol (TAP), florfenicol (FF), and florfenicol amine (FFA) in beef, pork, chicken, shrimp, eel, and flatfish using a quick, easy, cheap, effective, rugged, and safe (QuEChERS) extraction method coupled with liquid chromatography-tandem mass spectrometry (LC-MS/MS). Primary-secondary amine (PSA) and MgSO_4_ were used for sample purification. The analytes were separated on a reversed-phase analytical column. The coefficients of determination for the linear matrix-matched calibration curves were ≥0.9941. Recovery rates ranged between 64.26 and 116.51% for the four analytes with relative standard deviations (RSDs) ≤ 18.05%. The calculated limits of detection (LODs) and limits of quantification (LOQs) were 0.005–3.1 and 0.02–10.4 μg/kg, respectively. The developed method was successfully applied for monitoring samples obtained from local markets in Seoul, Republic of Korea. The target residues were not detected in any tested matrix. The designed method was versatile, sensitive, and proved suitable for quantifying residues in animal-derived products.

## Introduction

The increasing demand for meat products has led to an expansion in intensive animal farming. In 2018, global meat production reached 342 million tons, and fishery and aquaculture production reached 179 million tons (1). Factory farming exposes animals to higher levels of stress and a broader spectrum of diseases (2); thus, the use of antibiotics in animal farming has been steadily increasing (3). Worldwide, 73% of all antimicrobials (mainly antibiotics) are consumed by animals farmed for food (4). Hence, the accumulation of drug residues in edible tissues of animal and fish products is highly likely, which would pose a public health hazard, particularly for the consumers of those products (5). Previous studies have shown that approximately 4% of antimicrobial resistance formed in the human body has been transferred from animals (6). Amphenicols (chloramphenicol (CAP), thiamphenicol (TAP), florfenicol (FF), and florfenicol amine (FFA)) are among the antibiotics that cause resistance. Despite usage regulations, amphenicol antibiotics are widely used illegally by farmers of various animals due to their broad range of effects and low cost (7, 8). Therefore, the quantification of amphenicol antibiotic residual levels in meat and fish products is necessary.

CAP, TAP, FF, and FFA (Supplementary Figure 1) antibiotics belong to the family of amphenicols and are extensively administered to livestock to prevent and treat various infections due to their ability to inhibit the growth of both gram-positive and negative bacteria (9, 10). CAP, the first antibiotic isolated from *Streptomyces venezuelae* in 1947, binds to the 50S subunit of bacterial ribosomes and inhibits intra-bacterial protein synthesis (10, 11). This drug is highly effective in treating animal diseases; however, it exhibits many toxic effects. Its use is thus restricted in many countries, including the United States, the Republic of Korea, and those in the European Union (EU) (12–14). TAP and FF are structural analogs of CAP (15). FF is widely used to prevent and treat bacterial infections in livestock because its bioavailability in many species is considerably higher than tetracycline antibiotics (16, 17). Furthermore, it is used in the aquaculture industry to treat bacterial diseases (18). Following animal administration, FF is partially converted to FFA and florfenicol oxamic acid (19). FFA is a major metabolite of FF in beef, pork, and chicken (20). Therefore, in many countries, FFA has been designated as one of the marker residues indicative of FF presence (21).

Various analytical methods entailing LC-MS/MS have been reported for determining amphenicols in animal tissues following various extraction techniques, either single (22, 23) or multiple analytes (24–27). For example, CAP was extracted from seafood products, honey, and royal jelly using methanol by Kikuchi et al. (22). CAP, TAP, FF, and FFA were extracted from poultry, swine, bovine, and fish muscle using liquid-liquid extraction (LLE) (24). Fedeniuk et al. (27) quantified CAP, CAP 3-O-β-d-glucuronide (CAP-GLUC), TAP, FF, and FFA in the bovine, equine, and porcine liver following a modified quick, easy, cheap, effective, rugged, and safe (QuEChERS) extraction method combined with SPE cleanup. However, to the best of our knowledge, few studies have reported the use of the “QuEChERS” method for the simultaneous determination of CAP, TAP, FF, and FFA in a variety of food products (beef, pork, chicken, shrimp, eel, and flatfish).

Hence, the purpose of this study was to establish an accurate and sensitive method using modified QuEChERS extraction methods and LC-MS/MS for the quantification of CAP, TAP, FF, and FFA in commonly consumed products, including beef, pork, chicken, shrimp, eel, and flatfish in a single chromatographic run. This study was conducted based on the maximum residue limits (MRL) established by the Korean Ministry of Food and Drug Safety (KMFDS) and others (28–32) (Table 1). The MRL of FF in eel and flatfish (100 μg/kg) is higher than the quantifiable concentration range of the analytical device. Therefore, the reference concentration was lowered to 50 μg/kg. Analytes without a specified MRL were set at 10 μg/kg. The KMFDS has banned the use of CAP in animal products. Thus, it was analyzed based on the minimum required performance limit (MRPL) (0.5 μg/kg) of the KMFDS. The study adhered to the guidelines established by the Codex Alimentarius Commission (33).

**Table 1:** Maximum residue limit (MRL) and minimum required performance limit (MRPL) criteria for six animal-derived food products set by various regulatory authorities.

**Analytes**	**Matrix**	**KMFDS^1^ (μg/kg)**	**Codex^2^ (μg/kg)**	**JAP^3^ (μg/kg)**	**AUS^4^ (μg/kg)**	**EU^5^ (μg/kg)**	**USA^6^ (μg/kg)**
Chloramphenicol	Beef	0.5^a^	–	–	–	–	–
	Pork	0.5^a^	–	–	–	–	–
	Chicken	0.5^a^	–	–	–	–	–
	Shrimp	0.5^a^	–	–	–	–	–
	Eel	0.5^a^	–	–	–	–	–
	Flatfish	0.5^a^	–	–	–	–	–
Thiamphenicol	Beef	50	–	20	–	50	–
	Pork	50	–	–	–	50	–
	Chicken	50	–	50	–	50	–
	Shrimp	-^b^	–	–	–	50	–
	Eel	-^b^	–	–	–	50	–
	Flatfish	50	–	–	–	50	–
Florfenicol	Beef	100^c^	–	150	150	100	150
	Pork	150^c^	–	150	250	50	–
	Chicken	50^c^	–	100	–	50	–
	Shrimp	50^c^	–	–	–	50	–
	Eel	100^c^	–	4000	–	500	–
	Flatfish	100^c^	–	500	-	500	-
Florfenicol amine	Beef	100	–	150	150	100	150
	Pork	150	–	150	250	50	–
	Chicken	50	–	100	–	50	–
	Shrimp	50	–	–	–	50	–
	Eel	100^d^	–	4000	–	500	–
	Flatfish	100^d^	–	500	–	500	–

–* No MRL*.

a *The KMFDS has banned chloramphenicol use in farmed animals. It was tested based on MRPL (0.5 μg/kg)*.

b *Set to 10 μg/kg for analytes without a specified MRL*.

c *Set to 10 μg/kg for analytes with higher than the quantifiable concentration range of the device*.

d *This value is specified as 50 μg/kg, lower than the established MRL (100 μg/kg)*.

1 *Korean Ministry of Food and Drug Safety (2019) (14)*.

2 *Codex Alimentarius Commission (2018) (29)*.

3 *The Japan Food Chemical Research Foundation (2021) (31)*.

4 *Australian Pesticides and Veterinary Medicines Authority (2019) (28)*.

5 *European Commission (2010) (30)*.

6 *US Food and Drug Administration (2021) (32)*.

## Materials and Methods

### Chemicals, Reagents, and Samples

CAP (99.8% purity, CAS No.: 56-75-7), TAP (99.9% purity, CAS No.: 15318-45-3), FF (99% purity, CAS No.: 73231-34-2), FFA (99.3% purity, CAS No.: 76639-93-5), acetic acid (99.5% purity), ammonium hydroxide solution (NH_4_OH), and ethylenediaminetetraacetic acid disodium salt (EDTA) solution (0.5 M in H_2_O) were acquired from Sigma-Aldrich Corporation (St. Louis, MO, USA). HPLC-grade methanol (MeOH; 99.9% purity) and acetonitrile (ACN; 100% purity) were purchased from Pharmaco-Aaper (Brookfield, CT, USA) and JT Baker (Phillipsburg, NJ, USA). QuEChERS dSPE kits (containing 150 mg of primary-secondary amine (PSA) and 900 mg of MgSO_4_) were obtained from Phenomenex (Torrance, CA, USA). Cellulose acetate membrane filters (0.45 μm) were supplied by MILLEX (Merck Millipore Ltd, Co. Cork, Ireland), and 0.2 μm PTFE syringe filters were sourced from Pall Corporation (Michigan, USA). The polypropylene conical tubes (15 and 50 mL) used throughout the entire experiment were acquired from FALCON (Tamaulipas, Mexico). Ultrapure water (resistivity of 18.2 MΩ.cm at 25°C) was supplied by a Milli-Q water purification system (Millipore, Bedford, MA, USA). All matrices (beef, pork, chicken, shrimp, eel, and flatfish) were procured from local markets in Seoul, Republic of Korea.

### Preparation of the Standard Solutions

The primary stock standard solutions of CAP, TAP, and FF (1,000 μg/mL) in MeOH were prepared by reconstituting each drug standard according to its respective purity. The FFA stock solution (200 μg/mL) was prepared by accurately dissolving 2.0 mg of FFA in 1 mL double-distilled water (DDW) and 9 mL of ACN using an AG 285 analytical balance (Mettler Toledo, Seoul, Republic of Korea). The stock solutions were stored in the dark at −20°C and diluted accordingly before analysis. Depending on the levels of validation, the concentrations of the analyte working solutions differed. The mixed working solutions at specific CAP, TAP, FF, and FFA concentrations were prepared by diluting the stock solutions with ACN. All working standard solutions were stored at −20°C and analyzed within a week.

### Extraction Procedures

Homogenized beef, pork, chicken, shrimp, eel, and flatfish matrices (2.0 g) were weighed in 50 mL conical tubes. The samples were spiked with 0.2 mL of the working solution and equilibrated for 5 min. Next, a mixture of 1 mL of 0.1M EDTA in DDW and 1 mL of ammonium hydroxide: DDW (2:98, *v/v*) was added, and the mixture was vortex-mixed for 5 min. Then, 1% acetic acid in a 10 mL ACN was added to beef, pork, and chicken samples, while 10 mL ACN was added to shrimp, eel, and flatfish samples. After vortex-mixing for 10 min, the mixtures were sonicated in an ultrasonic bath (Bransonic 8210 ultrasonic cleaner, Branson Ultrasonics Corporation, Danbury, CT, USA) at 25 °C (40 k*Hz*: 5 min). The samples were centrifuged at 1392 rcf for 15 min at 4°C (Allegra X-15R, Beckman Coulter Inc., Brea, CA, USA), and the supernatants were transferred to tubes containing 150 mg PSA and 900 mg MgSO_4_. These mixtures were vortexed for 10 min and centrifuged at 1392 rcf for 15 min at 4 °C. The obtained supernatant was transferred to a clean 15 mL conical tube and dried under nitrogen gas at 40 °C using a TurboVap®RV device (Caliper Life Sciences, Hopkinton, USA) to remove all the moisture. Before analysis, the residues were reconstituted in 1 mL ACN: DDW (90:10, *v/v*), sonicated at 25 °C (40 k*Hz*: 5 min) and centrifuged at 12525 rcf at 4 °C for 15 min. Before LC-MS/MS analysis, the concentrated solutions were filtered through a 0.2 μm PTFE syringe filter and passed through a 0.22-μm filter before LC-MS/MS analysis.

### LC-MS/MS Instrumentation and Conditions

#### Chromatography Conditions

LC-MS/MS analysis was conducted using a Shimadzu high-performance liquid chromatography system (Columbia, MD, USA) equipped with two pumps (LC-30 AD), an autosampler (SIL-30AC), a degasser (DGU-20A5R), and a column oven (CTO-30A). Mass spectrometric detection was performed on a Shimadzu 8060 LC-MS/MS system (Shimadzu Scientific, Inc., MD, USA). Chromatographic separation was achieved on a Phenomenex Luna omega polar C18 100 Å (100 × 2.1 mm, 3 μm) at a column oven temperature of 40 °C and an injection volume of 3 μL. The mobile phases used for separating analytes were (A) DDW and (B) 0.1% acetic acid in ACN. The flow rate was 0.2 mL/min with a linear mobile phase gradient (time (min), % mobile phase B) at the following conditions: (0–1, 10% B); (1–2.5 min, 10%−100% B); (2.5–3.5 min, 100% B); (3.5–3.6 min, 100–10% B); (3.6–6 min, 10% B).

#### Mass Spectrometry Conditions

An electrospray ionization (ESI) in both positive (ESI+) and negative (ESI–) ion-switching modes were used for the triple quadrupole mass spectrometer (MS/MS). FFA was analyzed in positive ion mode, whereas CAP, TAP, and FF were analyzed in negative ion mode. Multiple reaction monitoring (MRM) mode and LabSolutions (version 5.89, Shimadzu) analyst software was implemented for data collection. The operating conditions of the mass spectrometer were as follows: an interface temperature of 300°C, heat block temperature of 400°C, dwell time of 17 ms, and ion spray voltage of ±3 kV. Individual working standard solutions (0.1 μg/mL) were employed for optimizing the precursor ion, product ion, and collision energy. The fragment [M+H]^+^ of the precursor ion was employed to identify FFA, whereas the [M–H]^−^ ion was selected for CAP, TAP, and FF. The MRM transitions and parameters are presented in Table 2.

**Table 2:** Multiple reaction monitoring (MRM) transitions of the tested drugs.

**Analytes**	**CAS No**.	**Molecular Weight**	**Precursor ion (*m/z*)**	**Identity**	**Product ion (*m/z*)**	**Q1 Pre Bias (V)**	**Collision Energy (eV)**	**Q3 Pre Bias (V)**
Chloramphenicol	56-75-7	323.1	321.1	[M-H]^−^	152.1^*^	12	17	15
					257.05	22	11	28
Thiamphenicol	15318-45-3	356.2	354.1	[M-H]^−^	185.1^*^	13	19	11
					290	13	12	12
Florfenicol	73231-34-2	358.2	356.1	[M-H]^−^	335.95^*^	13	9	10
					185.1	13	19	10
Florfenicol amine	76639-93-5	247.3	248.0	[M+H]^+^	230.1^*^	−15	−13	−25
					130.05	−14	−22	−22

* *Quantification ion*.

### Method Validation

The following parameters were validated according to KMFDS, 2019: linearity, precision (relative standard deviation, RSD), accuracy (recovery), limits of detection (LODs), and limits of quantification (LOQs). Four drugs were tested according to the specified MRL set by the KMFDS. Matrix-fortified calibration curves achieved linearity at six concentration levels based on Table 3. The calibration curves were constructed by plotting the response factor as a function of drug concentration. The calculated LOD and LOQ were obtained as signal-to-noise ratios of (S/N) ≥3 and S/N ≥10. Accuracies of intra-day (single day, *n* = 3) and inter-day (three days, *n* = 9) recovery values were estimated at three spiking levels (CAP: 0.5, 1, and 5 μg/kg; TAP, FF, and FFA: ×1/2, ×1, ×2 of the MRL values). The intra- and inter-day RSD were calculated at the above-listed concentrations. Additionally, matrix effects at a spiking level of 0.5 μg/kg (CAP) and 1MRL values (TAP, FF, and FFA) were calculated for all four amphenicols.

**Table 3:** Method performance for chloramphenicol, thiamphenicol, florfenicol, and florfenicol amine analysis in spiked beef, pork, chicken, shrimp, eel, and flatfish samples.

**Analytes**	**Matrix**	**Spiking level (μg/kg)**	**Intra-day (*n* = 3)**	**Inter-day (*n* = 9)**	***R^2^***	**LOD (μg/kg)**	**LOQ (μg/kg)**
			**Recovery (RSD) (%)**	**Recovery (RSD) (%)**			
Chloramphenicol	Beef	0.5	83.61 (7.80)	97.90 (1.05)	0.9980	0.01	0.04
		1	94.66 (5.03)	115.49 (3.26)			
		5	101.12 (1.20)	107.17 (4.36)			
	Pork	0.5	94.66 (4.24)	95.07 (7.69)	0.9994	0.01	0.04
		1	106.76 (7.77)	102.14 (4.93)			
		5	99.73 (6.53)	87.46 (4.38)			
	Chicken	0.5	92.30 (14.23)	106.01 (12.79)	0.9971	0.02	0.07
		1	109.32 (5.21)	116.51 (3.05)			
		5	108.82 (4.90)	94.31 (7.24)			
	Shrimp	0.5	84.34 (11.53)	77.85 (6.70)	0.9989	0.01	0.05
		1	92.14 (5.33)	75.02 (3.14)			
		5	74.54 (15.73)	64.26 (7.78)			
	Eel	0.5	95.48 (9.71)	84.18 (4.41)	0.9990	0.01	0.04
		1	92.41 (11.33)	98.78 (1.12)			
		5	79.66 (8.29)	82.73 (1.03)			
	Flatfish	0.5	92.50 (4.53)	91.24 (7.03)	0.9976	0.01	0.02
		1	99.18 (2.52)	86.91 (4.63)			
		5	81.90 (9.45)	81.95 (2.92)			
Thiamphenicol	Beef	25	71.66 (3.11)	86.86 (2.86)	0.9993	0.1	0.3
		50	93.69 (8.28)	99.68 (3.92)			
		100	97.06 (5.14)	96.65 (1.48)			
	Pork	25	73.20 (3.57)	74.37 (2.28)	0.9994	0.09	0.3
		50	100.36 (7.19)	83.59 (4.23)			
		100	95.22 (3.30)	78.48 (6.04)			
	Chicken	25	96.62 (3.38)	87.38 (3.53)	0.9996	0.1	0.3
		50	100.74 (6.46)	92.79 (7.89)			
		100	91.57 (8.27)	91.30 (2.34)			
	Shrimp	5	91.20 (1.37)	88.85 (4.68)	0.9990	0.09	0.3
		10	86.66 (7.92)	84.69 (6.88)			
		20	80.32 (10.35)	83.34 (4.87)			
	Eel	5	96.51 (7.03)	96.92 (4.45)	0.9996	0.05	0.2
		10	86.25 (6.79)	99.59 (6.47)			
		20	81.19 (9.46)	83.36 (4.47)			
	Flatfish	25	90.92 (7.46)	92.22 (4.19)	0.9989	0.05	0.2
		50	106.91 (6.51)	96.67 (4.47)			
		100	106.41 (9.51)	104.39 (5.13)			
Florfenicol	Beef	5	93.91 (5.05)	76.12 (5.21)	0.9989	0.01	0.04
		10	100.50 (4.21)	87.20 (1.68)			
		20	102.47 (1.90)	86.82 (2.40)			
	Pork	5	104.43 (2.40)	102.82 (4.49)	0.9995	0.02	0.06
		10	100.88 (6.15)	104.03 (2.43)			
		20	90.89 (2.80)	97.08 (1.96)			
	Chicken	5	93.60 (11.08)	96.25 (8.38)	0.9995	0.01	0.02
		10	90.77 (10.92)	105.49 (6.71)			
		20	96.50 (2.11)	109.57 (0.81)			
	Shrimp	5	84.75 (11.31)	81.54 (8.35)	0.9976	0.01	0.04
		10	101.92 (3.05)	92.98 (3.84)			
		20	91.17 (6.15)	88.30 (11.49)			
	Eel	5	85.54 (7.90)	90.96 (3.53)	0.9992	0.01	0.02
		10	90.93 (5.26)	99.04 (2.12)			
		20	86.94 (5.17)	86.06 (2.26)			
	Flatfish	5	85.30 (6.34)	85.05 (6.13)	0.9980	0.005	0.02
		10	101.17 (4.79)	90.05 (5.56)			
		20	100.13 (5.59)	96.36 (6.35)			
Florfenicol amine	Beef	50	93.11 (6.38)	80.72 (5.52)	0.9941	3.1	10.4
		100	88.46 (5.87)	85.34 (3.44)			
		200	86.46 (4.35)	81.29 (2.65)			
	Pork	75	88.66 (1.78)	82.95 (1.50)	0.9998	1.5	5.1
		150	82.12 (13.16)	74.70 (1.19)			
		300	94.28 (9.65)	84.43 (1.19)			
	Chicken	25	78.08 (2.58)	85.37 (2.27)	0.9964	0.6	1.8
		50	88.16 (18.05)	91.58 (14.39)			
		100	87.45 (1.47)	83.01 (1.50)			
	Shrimp	25	93.72 (6.32)	100.57 (2.62)	0.9991	1.3	4.3
		50	101.23 (1.34)	91.71 (4.76)			
		100	91.05 (4.46)	85.49 (5.83)			
	Eel	25	89.39 (1.94)	91.22 (0.69)	0.9979	2.1	7.1
		50	92.72 (4.31)	107.36 (2.47)			
		100	76.29 (12.65)	82.41 (3.57)			
	Flatfish	25	92.64 (5.65)	91.86 (3.47)	0.9979	1	3.3
		50	89.06 (10.50)	82.97 (9.23)			
		100	86.64 (5.38)	86.45 (2.93)			

### Statistical Analyses

IBM SPSS Statistics, v25 (NY, USA) was used to compare the various extraction methods. Statistical analysis was performed using one-way ANOVA. Differences were considered significant when *P* ≤ 0.05.

## Results and Discussion

### Optimization of Sample Preparation

**Figure 1:**
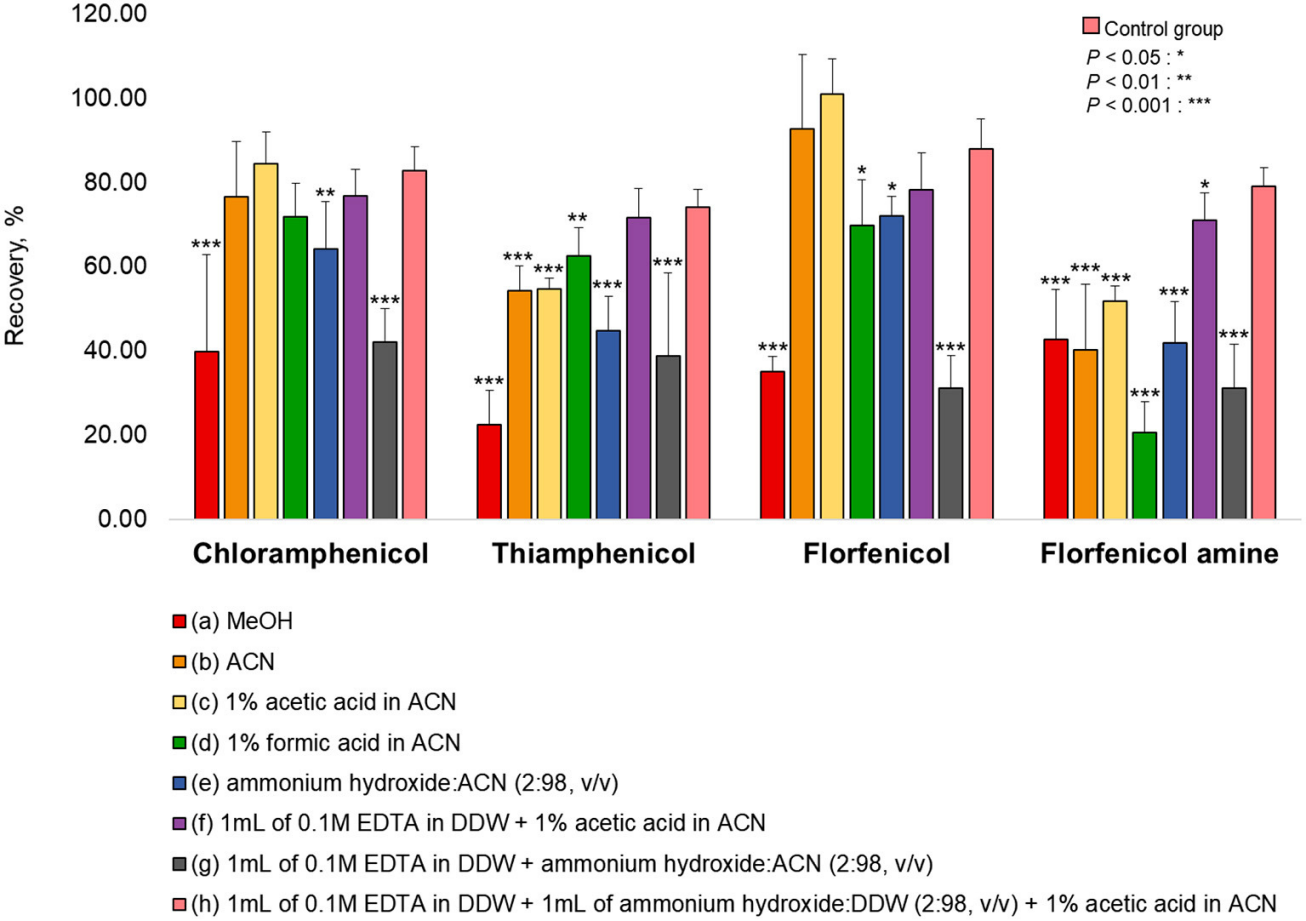
Extraction efficiencies of various solvents for the tested analytes in pork. The pork was fortified at a concentration rate of CAP: 1 μg/kg, TAP: 50 μg/kg, FF: 10 μg/kg, and FFA: 150 μg/kg for each extraction protocol. (h) was used as a control group. Statistical analysis (IBM SPSS Statistics, v25, NY, USA) was conducted using one-way ANOVA analysis. *P < 0.05; **P < 0.01; and ***P < 0.001 were considered statically significant.

**Figure 2:**
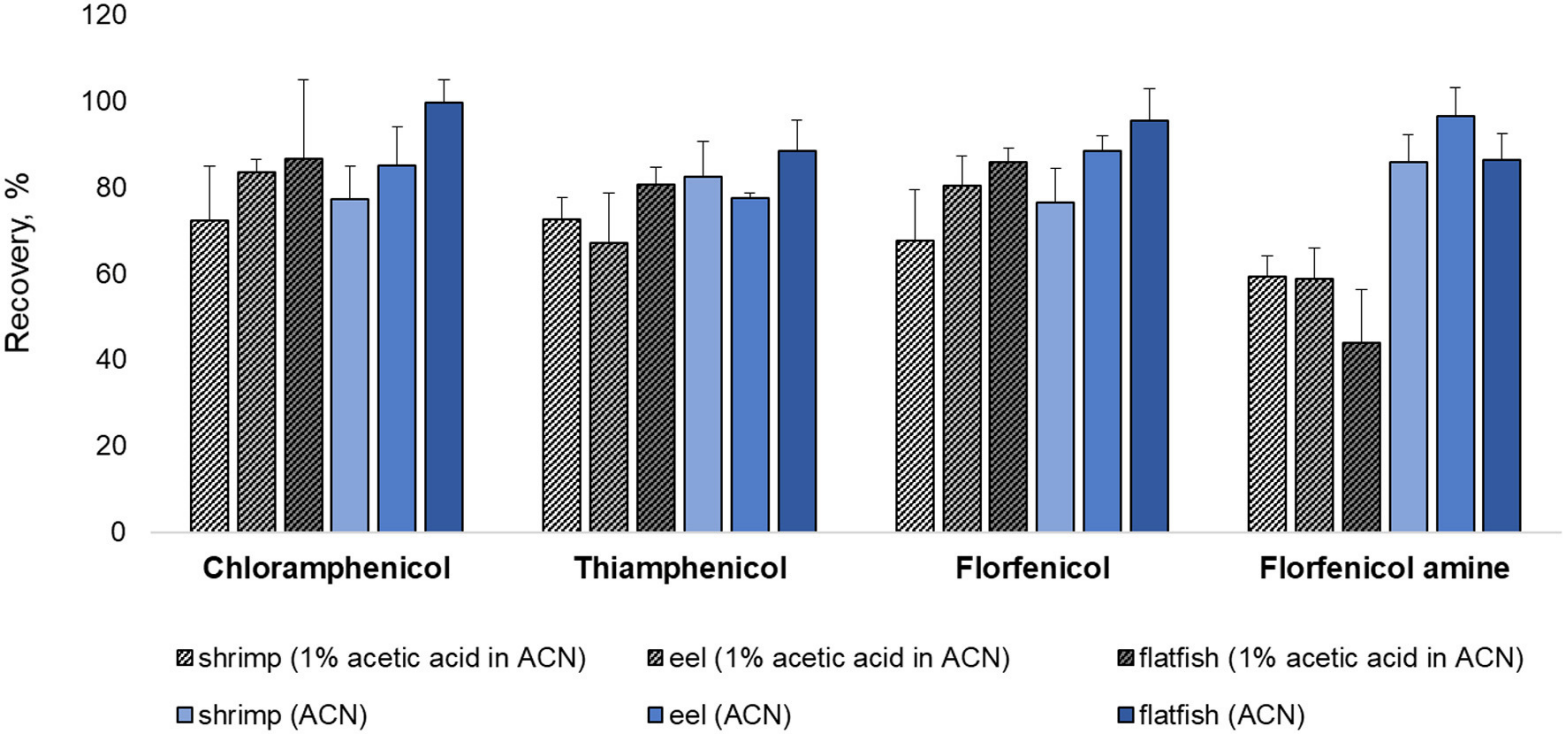
Comparison of extraction methods with or without 1% acetic acid addition for shrimp, eel, and flatfish. Three samples were fortified at their respective MRL (CAP: MRPL) concentrations.

Various extraction methods were conducted to optimize the sample preparation protocol and achieve the maximum extraction efficiency (recovery rate). As a representative of all the samples, pork was fortified at a concentration rate based on the MRL (CAP: MRPL). The extraction of chopped samples was evaluated in ACN containing acid or base and in MeOH (common extraction solvents used for protein precipitation. The eight extraction conditions were: (a) MeOH, (b) ACN, (c) 1% acetic acid in ACN, (d) 1% formic acid in ACN, (e) ammonium hydroxide: ACN (2:98, *v/v*), (f) 1 mL of 0.1M EDTA in DDW + 1% acetic acid in ACN, (g) 1 mL of 0.1M EDTA in DDW + ammonium hydroxide: ACN (2:98, *v/v*), (h) 1 mL of 0.1 M EDTA in DDW + 1 mL of ammonium hydroxide: DDW (2:98, *v/v*) + 1% acetic acid in CAN (26, 27, 34–37). The recovery rates for each condition are shown in Figure 1. The recovery rate of FFA using (h) (79.13%) was 10%−60% higher than achieved under other conditions, which were: (a) 42.82%, (b) 40.21%, (c) 51.86%, (d) 20.56%, (e) 41.95%, (f) 70.99%, and (g) 31.23%. Furthermore, employing (h), the recovery rates of CAP, TAP, and FF were 82.76, 74.16, and 87.99 %, respectively. However, conditions (h) did not provide a satisfactory recovery rate of FFA from fishery products (shrimp: 59.26%, eel: 58.79%, and flatfish: 43.93%). The use of ACN (without acetic acid) as an extraction solvent increased the recovery rate of shrimp (85.81%), eel (96.55%), and flatfish (86.42%) (Figure 2). Therefore, the selected extraction solvent for beef, pork, and chicken was 0.1 M EDTA in DDW + ammonium hydroxide: DDW (2:98, *v/v*) + 1% acetic acid in ACN and 0.1 M EDTA in DDW + ammonium hydroxide: DDW (2:98, *v/v*) + ACN for shrimp, eel, and flatfish.

Livestock products contain a higher proportion of endogenous interferences, such as lipids, phospholipids, and fatty acids, than vegetables and fruits; a purification process is necessary (35, 37). Therefore, four purification protocols were compared, namely: (A) 10 mL of *n*-hexane saturated with ACN, (B) C18 150 mg, (C) PSA 150 mg, C18 150 mg, and MgSO_4_ 900 mg, and (D) PSA 150 mg and MgSO_4_ 900 mg. Recoveries ranging from 71.91 to 89.49% (RSD: 4.80–9.18%), 60.96–82.08% (RSD: 6.52–9.25%), 68.40–88.97% (RSD: 1.64–8.92%), and 79.08–97.52% (RSD: 0.57–3.57%) were obtained under the conditions of (A), (B), (C), and (D), respectively. The recovery rate of condition (D) was the highest with the lowest RSD; thus, it was chosen as the purification method. Then, a high-speed centrifuge was used to remove low-layer impurities and obtain a clearer solution. Additionally, a syringe filter was used for further purification and instrument protection.

### LC-MS/MS Conditions

The amphenicol drugs were analyzed using ESI turbo-positive and negative ion modes (positive ion mode: FFA; negative ion mode: CAP, TAP, and FF). All parameters used for quantification in MRM mode are shown in Table 2. For LC-MS/MS analysis, several combinations of mobile phases (A) and (B) were tested due to the significant effect of mobile phase composition on ionization efficiency. The solvents tested in the mobile phase (A) were: DDW, 0.1% acetic acid, 0.2% acetic acid, 0.1% formic acid, 1 mM ammonium formate, and 1 mM ammonium acetate. When acid and ammonium were not added to DDW, the peak intensities were satisfactory, and the peak of FFA was sharp. For solvent (B), ACN, 0.1% acetic acid in ACN, 0.1% formic acid in ACN, and MeOH were compared. When ACN or MeOH was used, peak tailing and peak splitting were observed for CAP, and a messy baseline and weak peak sensitivity were noted for all the drug analytes. ACN with 0.1% acetic acid was chosen as solvent (B) as it provided the sharpest peak shape and high-intensity peaks. Various C18 columns based on a silica hydride support, including basic (such as Phenomenex Luna C18 and Phenomenex Kinetex C18), high-strength silica (such as Waters X-Select HSS C18), extensive pH (such as Phenomenex Kinetex EVO C18), consistent performance in both volatile and non-volatile buffers (such as Phenomenex Gemini-NX C18), and polarity (such as Phenomenex Luna omega polar C18) were compared to obtain optimal peak parameters for the four tested drugs. The use of columns with basic, high-intensity silica, and extensive pH characteristics led to peak broadening/splitting. The Phenomenex Gemini-NX C18 column with consistent performance in volatile and non-volatile buffers gave poor signals and peak tailing for TA and FFA. Hence, the Phenomenex Luna omega polar C18 column (100 × 2.0 mm, 3 μm particle size) with unique polar selectivity was chosen to achieve optimal chromatographic separation.

### Method Performance

#### Specificity and Linearity

Specificity was determined by analyzing blank beef, pork, chicken, shrimp, eel, and flatfish samples (*n* = 3) spiked with each analyte at a concentration of the MRL values. As shown in Supplementary Figures 2, 3, no interference from endogenous materials was observed.

According to MFDS guidelines (14), matrix-matched calibration curves from the responses of the four drugs were constructed by plotting the peak area of each tested analyte *vs*. the concentration (CAP: ×1, ×2– ×6 the MRPL values; TAP, FF, and FFA: ×1/2, ×1, and ×2 to ×5 the MRL values, *n* = 3). The linearity was satisfactory, with coefficients of determination (*R*^2^) being ≥0.9941 for all matrices.

### LODs, LOQs, and Matrix Effects

As shown in Table 3, the LOD ranges were 0.01–0.02, 0.05–0.1, 0.005–0.02, and 0.6–3.1 μg/kg, and the LOQ ranges were 0.02–0.07, 0.2–0.3, 0.02–0.06, and 1.8–10.4 μg/kg for CAP, TAP, FF, and FFA, respectively. Furthermore, the LODs and LOQs were lower than the MRLs established for each drug. The LOQ of FFA was similar or higher than that in other studies (25, 27); however, the values for the other tested analytes were generally lower.

The matrix effects (MEs) gave rise to either ion suppression or enhancement depending on the matrix. MEs were determined at a spiking level of 0.5 μg/kg (CAP) and 1MRL values (TAP, FF, and FFA) as follows:


$$\begin{array}{l} \text{MEs}\left(\%\right)=\left(\text{A}-\text{B}\right)/\text{B}\times 100 \end{array}$$

where A denotes the peak area of the standard in the matrix and B denotes the peak area of the standard in pure solvent. ME ranges were: −70.17 – 11.97% (beef), −67.60 – −7.20% (pork), −68.74 – 9.22% (chicken), −89.20 – 18.92% (shrimp), −84.68 – 3.88% (eel), and −69.76 – 5.59% (flatfish). In general, matrices containing proteins and lipids exhibit significant matrix-specific effects (38). As livestock products contain numerous proteins and fat, matrix-specific effects could not be completely ruled out (39).

### Accuracy and Precision

The accuracy (expressed as recovery %) and precision (expressed as RSD%) were evaluated based on the criteria set by the Codex Alimentarius Commission (spiking concentrations: ≤ 1, 1–10, 10–100 and 100–1,000 μg/kg; recoveries: 50–120%, 60–120%, 70–120%, and 70–110%; RSDs: ≤ 35%, ≤ 30%, ≤ 20%, and ≤ 15% for CAP, TAP, FF, and FFA, respectively) (33). Blank samples spiked at three concentration levels (CAP: ×1, ×2, and ×6 the MRPL values; TAP, FF, and FFA: ×1/2, ×1, and ×2 the MRL values) were analyzed (*n* = 3) on a single day (intra-day) or for three consecutive days (inter-day) (*n* = 9). As shown in Table 3, the intra-day recovery values and RSDs were 71.66–109.32% and ≤ 18.05%, while the inter-day recovery values and RSDs were 64.26–116.51% and ≤ 14.39% for the four tested drugs in various matrices. These results show that the developed method satisfies the Codex guidelines.

### Comparison With Other Methods

As shown in Table 4, most of the studies employed LLE methods for extracting CAP, TAP, FF, and FFA. It has to be noted that only one study used the QuEChERS method; however, the recovery rate was lower than the present study. In addition, none of the studies monitored the analytes in various livestock and fishery products (beef, pork, chicken, shrimp, eel, and flatfish).

**Table 4:** Comparison with other studies. Matrices, extraction methods, analytical devices, recovery rates, LODs, and LOQs were compared for the tested drugs.

**No**.	**Analytes**	**Matrix**	**Extraction method**	**Analytical device**	**Recovery (RSD) %**	**LOD (μg/kg)**	**LOQ (μg/kg)**	**Reference**
1	Chloramphenicol, chloramphenicol 3-O-β-d-glucuronide, florfenicol, florfenicol amine and thiamphenicol	Bovine, equine, and porcine liver	Modified QuEChERS	LC-MS/MS	50–105 (7.6–45)	0.03–0.84	0.11–2.75	Fedeniuk et al. (27)
2	Chloramphenicol, thiamphenicol, florfenicol and florfenicol amine	Poultry, swine, bovine, and fish	LLE	LC-MS/MS	82–111 (1.1–18.1)	0.06–252.10	0.11–304.20	Barreto et al. (24)
3	Chloramphenicol, thiamphenicol, florfenicol, and florfenicol amine	Poultry eggs	LLE	UPLC-MS/MS	90.31–107.79 (1.42–6.65)	0.03–0.4	0.08–1.2	Wang et al. (25)
4	Chloramphenicol, thiamphenicol, florfenicol and florfenicol amine	Chicken muscle	LLE and SPE	LC-MS/MS	95.1–107.3 (4.4–10.9)	0.1–1	0.3–3	Zhang et al. (26)
5	Chloramphenicol, thiamphenicol, and florfenicol	Fish muscle	MSPD	UPLC-MS/MS	84.2–99.8 (<12)	-	-	Pan et al. (40)
6	Chloramphenicol, thiamphenicol, and florfenicol	Bovine muscle	tetrahydrofuran (THF)–water	LC-MS/MS	90–112 (5–15)	-	0.141–12.9	Sichilongo et al. (41)
7	Chloramphenicol, thiamphenicol, florfenicol, and florfenicol amine	Beef, pork, chicken, shrimp, eel, and flatfish	Modified QuEChERS	LC-MS/MS	64.26–116.51 ( ≤ 18.05)	0.005–3.1	0.02–10.4	This study

*SPE, solid-phase extraction; MSPD, Matrix Solid-Phase Dispersion Extraction; UPLC-MS/MS, ultra-performance liquid chromatography-tandem mass spectrometry*.

### Method Application

Commercial samples of beef, pork, chicken, shrimp, eel, and flatfish were obtained from local markets in Seoul, Republic of Korea. The samples were handled according to the method described in Section 2.3. As shown in Supplementary Figures 2, 3, none of the samples tested positive for the target analytes.

## Conclusions

An analytical protocol based on LC-MS/MS was developed and validated to simultaneously determine CAP, TAP, FF, and FFA. The four analytes were extracted from six samples (beef, pork, chicken, shrimp, eel, and flatfish) using LLE and modified QuEChERS methods for LC-MS/MS analysis. Recovery rate ranges of 64.26–116.51%, 71.66–106.91%, 76.12–109.57%, and 74.70–107.36% were obtained for CAP, TAP, FF, and FFA, respectively, in all matrices. The developed protocol offers a rapid and straightforward method for the simultaneous determination of these four analytes. Regulatory authorities can evaluate it as a reference method for establishing amphenicol MRLs in various livestock products.

## Data Availability Statement

The original contributions presented in the study are included in the article/Supplementary Material, further inquiries can be directed to the corresponding authors.

## Author Contributions

H-NJ and D-HP: formal analysis, investigation, validation, and writing-original draft. Y-JC and S-HK: methodology. H-JC: methodology and visualization. J-MC: methodology. J-HS and AZ: writing-review and editing. AA: data curation, formal analysis, and writing-review and editing. H-CS: conceptualization, resources, writing-review and editing, funding acquisition, project administration, and supervision. All authors contributed to the article and approved the submitted version.

## Funding

The study was supported by the Ministry of Food and Drug Safety Administration, Republic of Korea [grant number 20162MFDS621] in 2020.

## Conflict of Interest

The authors declare that the research was conducted in the absence of any commercial or financial relationships that could be construed as a potential conflict of interest.

## Publisher's Note

All claims expressed in this article are solely those of the authors and do not necessarily represent those of their affiliated organizations, or those of the publisher, the editors and the reviewers. Any product that may be evaluated in this article, or claim that may be made by its manufacturer, is not guaranteed or endorsed by the publisher.
