# Supplementary material for: Simultaneous Quantification of Chloramphenicol, Thiamphenicol, Florfenicol, and Florfenicol Amine in Animal and Aquaculture Products Using Liquid Chromatography-Tandem Mass Spectrometry
Source: Front Nutr. 2022 Jan 13;8:812803. doi: 10.3389/fnut.2021.812803 (PMC8793773; doi:10.3389/fnut.2021.812803)
Supplement: Supplementary file 1 [file Data_Sheet_1.PDF]

<u>Supplementary materials</u>

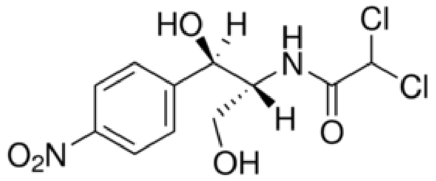

Chloramphenicol

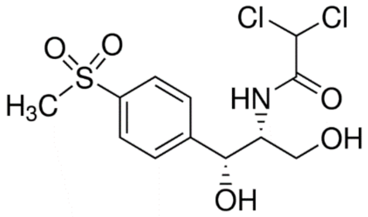

Thiamphenicol

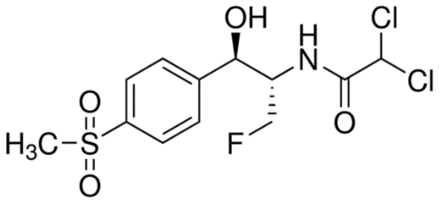

Florfenicol

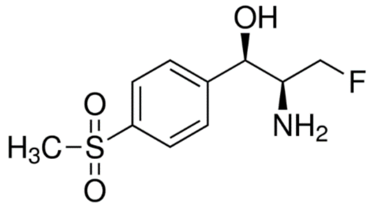

Florfenicol amine

Suppl. Figure 1. Chemical structures of chloramphenicol (CAP), thiamphenicol (TAP), florfenicol (FF), and florfenicol amine (FFA).

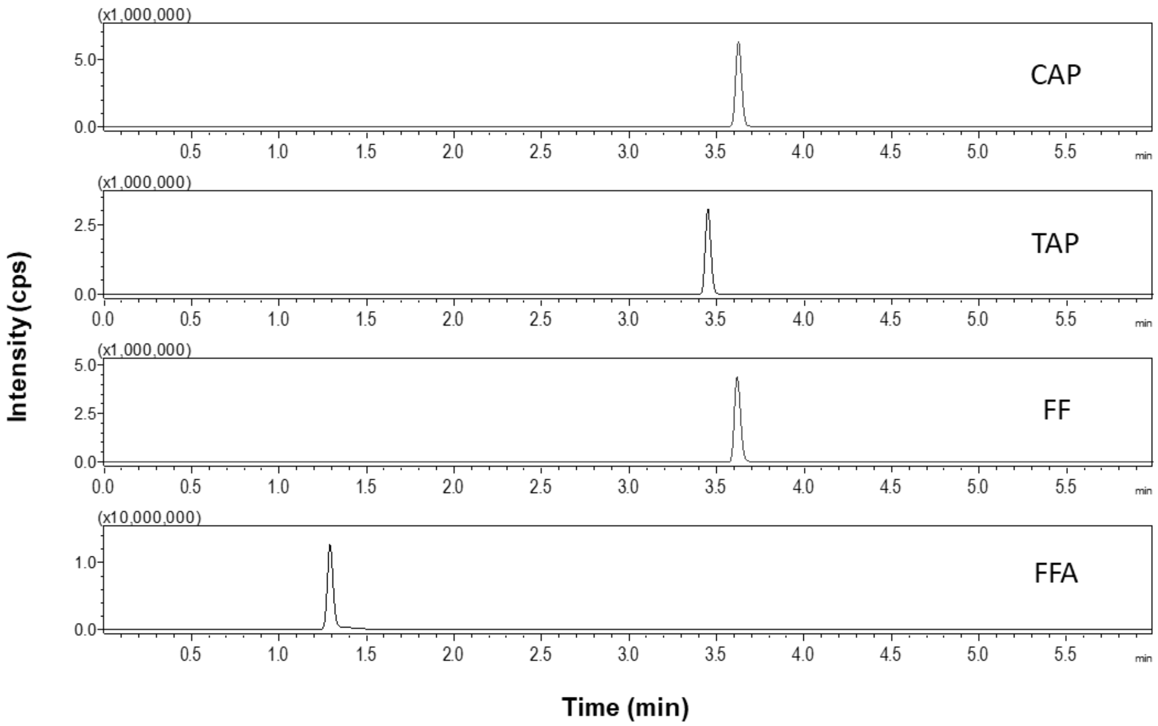

Suppl. Figure 2. LC-MS/MS chromatograms of the CAP, TAP, FF, and FFA standards (100 µg/kg).

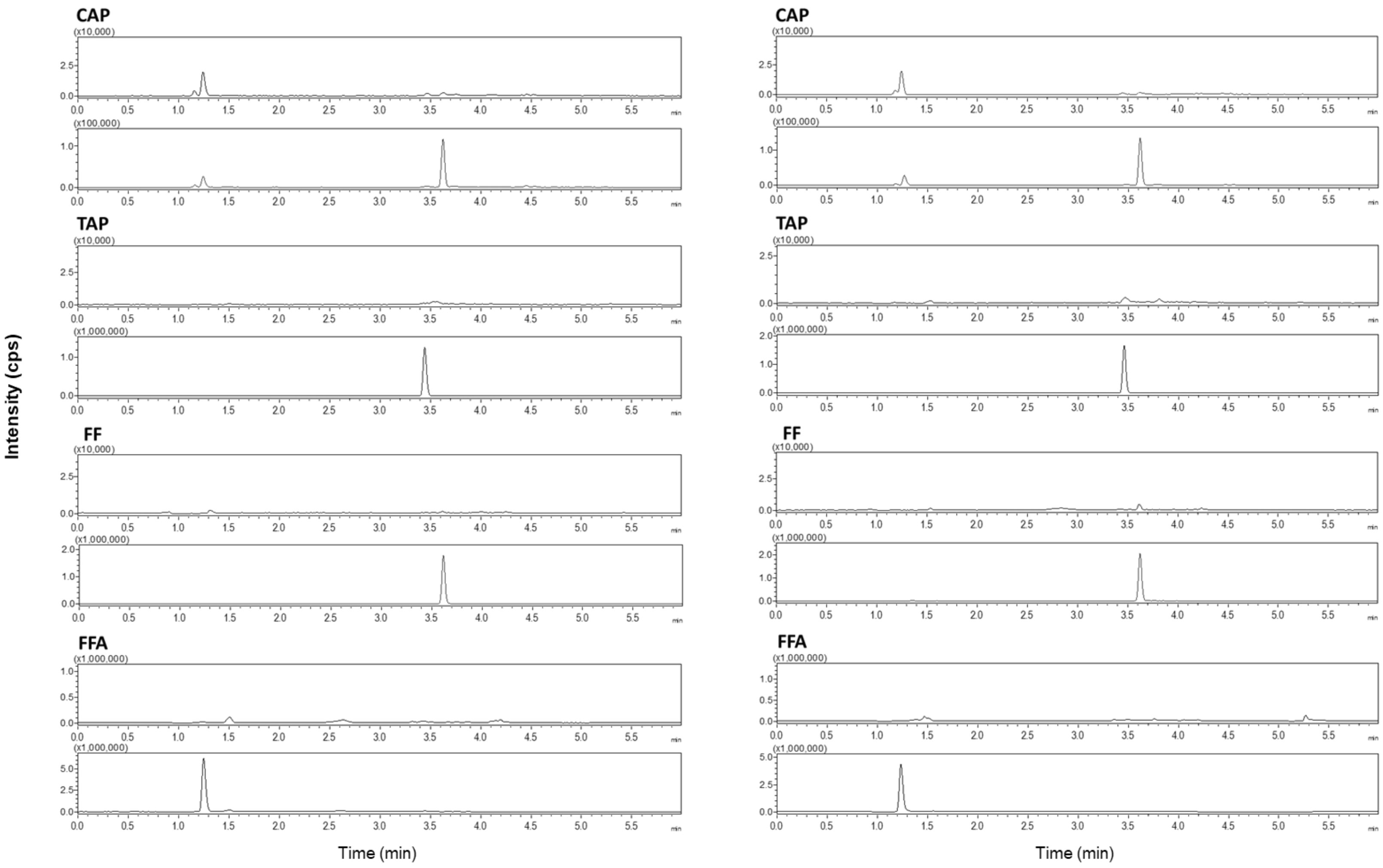
CAP
TAP
FF
FFA
Intensity (cps)
Time (min)
(x10,000)
(x100,000)
(x1,000,000)
min

(A) (B)

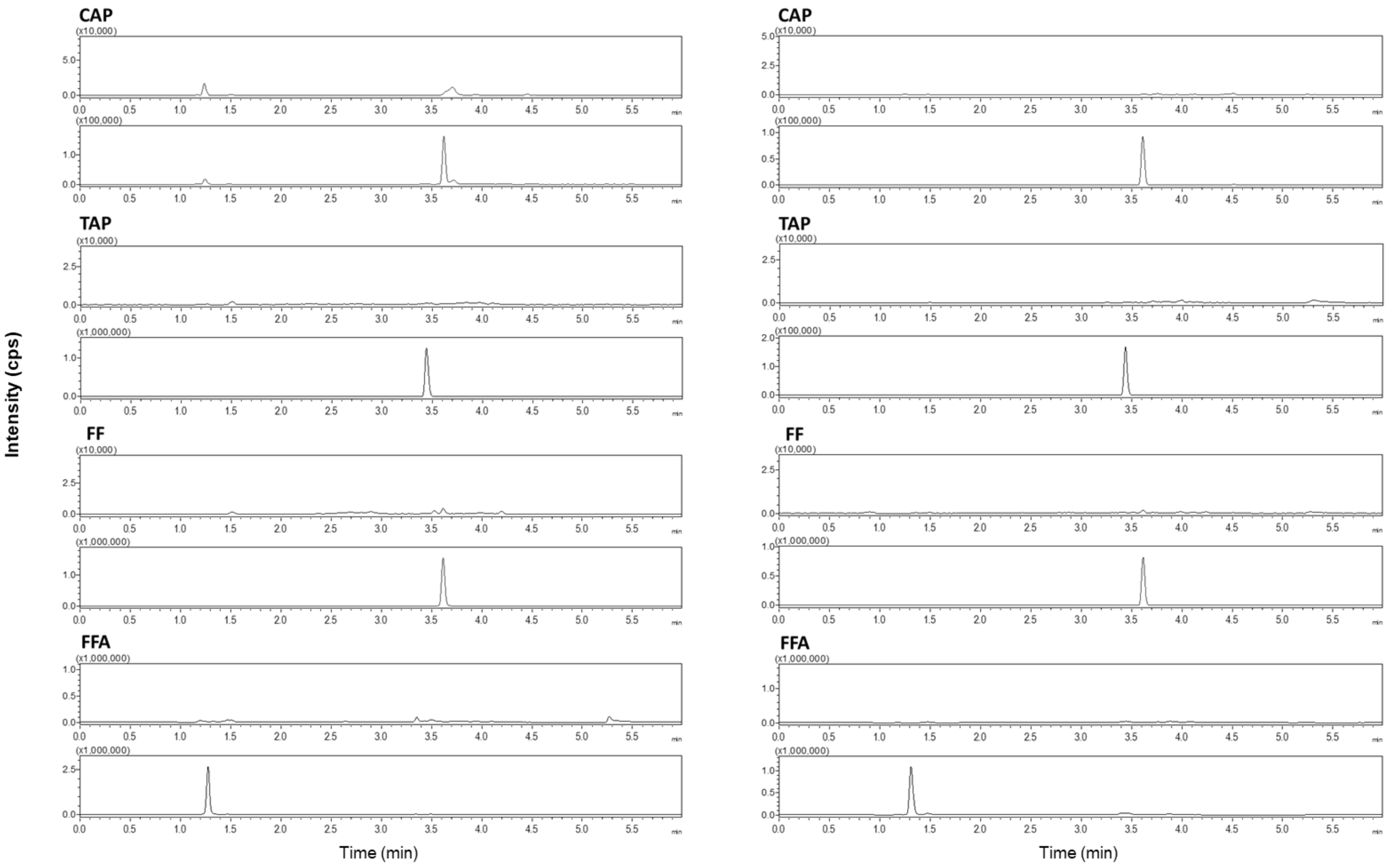

(C) (D)

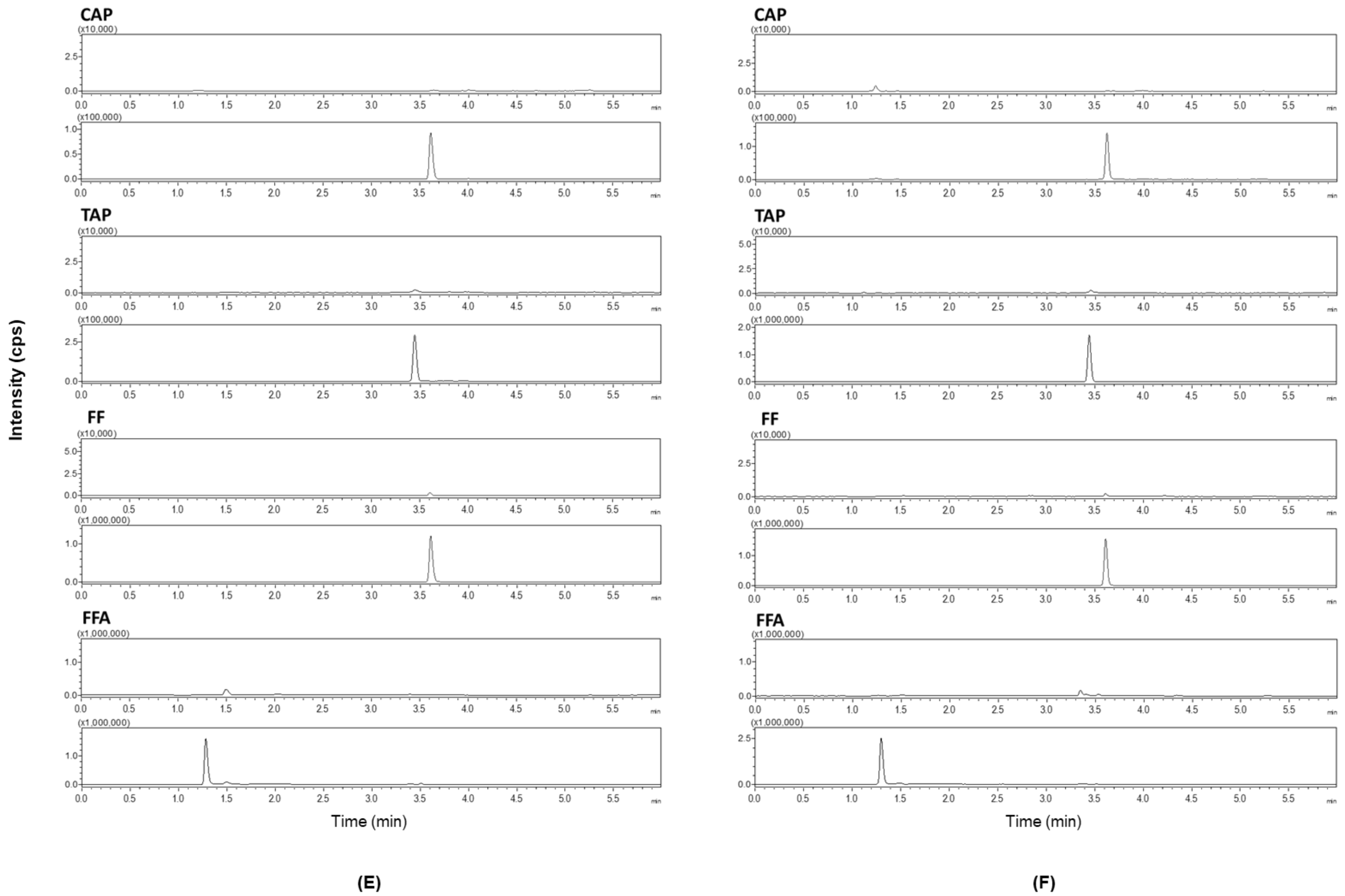

Suppl. Figure 3. LC-MS/MS chromatograms of the tested drugs in blank samples (top), spiked samples (bottom) of (A) beef, (B) pork, (C) chicken, (D) shrimp, (E) eel, (F) flatfish. All samples are fortified at their respective MRL (CAP: MRPL) concentrations.
